# Bio-Based Polyurethane Networks Derived from Liquefied Sawdust

**DOI:** 10.3390/ma14113138

**Published:** 2021-06-07

**Authors:** Kamila Gosz, Agnieszka Tercjak, Adam Olszewski, Józef Haponiuk, Łukasz Piszczyk

**Affiliations:** 1 Department of Polymers Technology, Chemical Faculty, Gdansk University of Technology, G. Narutowicza Str., 11/12, 80233 Gdansk, Poland; kamila.gosz@pg.edu.pl (K.G.); adam.olszewski@pg.edu.pl (A.O.); jozhapon@pg.edu.pl (J.H.); 2 Group ‘Materials + Technologies’ (GMT), Department of Chemical and Environmental Engineering, Engineering School of Donostia-San Sebastian, University of Basque Country (UPV/EHU), Plaza Europa 1, 20018 Donostia-San Sebastian, Spain; agnieszka.tercjaks@ehu.eus

**Keywords:** liquefaction, liquefied wood, bio-polyols, polyurethane resin

## Abstract

The utilization of forestry waste resources in the production of polyurethane resins is a promising green alternative to the use of unsustainable resources. Liquefaction of wood-based biomass gives polyols with properties depending on the reagents used. In this article, the liquefaction of forestry wastes, including sawdust, in solvents such as glycerol and polyethylene glycol was investigated. The liquefaction process was carried out at temperatures of 120, 150, and 170 °C. The resulting bio-polyols were analyzed for process efficiency, hydroxyl number, water content, viscosity, and structural features using the Fourier transform infrared spectroscopy (FTIR). The optimum liquefaction temperature was 150 °C and the time of 6 h. Comprehensive analysis of polyol properties shows high biomass conversion and hydroxyl number in the range of 238–815 mg KOH/g. This may indicate that bio-polyols may be used as a potential substitute for petrochemical polyols. During polyurethane synthesis, materials with more than 80 wt% of bio-polyol were obtained. The materials were obtained by a one-step method by hot-pressing for 15 min at 100 °C and a pressure of 5 MPa with an NCO:OH ratio of 1:1 and 1.2:1. Dynamical-mechanical analysis (DMA) showed a high modulus of elasticity in the range of 62–839 MPa which depends on the reaction conditions.

## 1. Introduction

Polyurethanes (PU) are one of the most important and most versatile classes of polymeric materials for a wide range of applications, i.e., flexible or rigid foams [1,2,3], thermoplastic elastomers [4], adhesives [5], coatings [6,7,8] and resins [9,10]. The production of polyurethanes is based on the polyaddition reaction between oligomeric polyols terminated with hydroxyl groups and diisocyanate. Due to the variety of oligomeric polyols, polyurethane structure and its end properties can be modulated. Polyols and isocyanate are the two main raw materials for polyurethane production and are currently obtained from fossil resources. The main disadvantage of petroleum products is that they are non-renewable and non-biodegradable. Due to human activity in the field of mass production, excessive consumption, and huge amounts of waste, environmental problems have been very serious since the beginning of the 20th century [11,12]. Therefore, the search for biomass-based alternatives has attracted a lot of attention. Particularly noteworthy is biomass obtained from forestry industry residues that are not fully utilized. It is also referred to as dry plant matter (i.e., waste wood). It consists of three main components, namely cellulose (30–50%), hemicellulose (15–35%), and lignin (20–35%), which contain two or more hydroxyl groups in the molecule and form a stable three-dimensional network structure [13,14]. Many types of agriculture and forestry industry biowastes have been used to create polyols. These biowastes include wood [2,9,15,16], wheat straw [4,17,18], corn bran [19], sugar cane [20], and bamboo [21], which can be treated as rapidly renewable bio-resources. Liquefaction of biomass in solvents is an effective method of converting it from solid to liquid [22].

Wood liquefaction can be carried out in the presence of phenol or polyhydric alcohols under acidic or alkaline conditions, and even without the use of a catalyst at higher temperatures [23]. Liquefied wood is a polyhydric mixture, rich in hydroxyl groups, which consists of biomass fragments, free polyhydric alcohol, and other by-products. Daneshvar et al. [24] carried out liquefaction of beech sawdust in the presence of ethylene carbonate and sulfuric acid at a temperature of 110 to 160 °C. The obtained bio-polyol was used for two types of polyurethane adhesive cross-linked at different NCO to OH ratios. Zhang et al. [25] optimized the process of liquefying agricultural waste to obtain polyurethane bio-foams. To this end, they used rice straw, rape straw, wheat straw, and corn shoots. Polyethylene glycol and ethylene glycol were the solvents while sulfuric acid was used as a catalyst. Agricultural waste was transformed with a high conversion rate of about 95% and the resulting bio-polyols showed suitable properties for obtaining bio-composites. Prepared polyurethane bio-foams showed comparable properties to materials obtained from petrochemical polyols. Lee et al. [15] showed that liquefied wood from *Cryptomeria japonica D* in ethylene glycol and sulfuric acid can be used as a raw material for the preparation of polyurethane resins which showed better mechanical properties and higher thermal resistance than materials made of polyester polyol. Pan et al. [26] used microwave radiation in the process of liquefying pine wood with a two-component solvent system-mixture of polyethylene glycol and glycerol. They showed that the bio–polyol has an appropriate hydroxyl value for the preparation of rigid PU foams. The materials obtained generally had lower compressive strength and apparent modulus than the control sample based on petroleum but showed better capability after deformation. This study aims to develop a new and ecological way of bio-polyol synthesis in the biomass liquefaction process. Implementation of such polyols can reduce the negative impact of the polyurethane industry on the environment. Moreover, this work aims to validate using bio-based polyols in the production of polyurethane resins. These resins can be applied as decking, tabletops, laminates, and panels.

In this study, the process of liquefying sawdust from oak *Quercus robur* (O) and alder *Alnus* (A) was carried out in the presence of glycerol, a mixture of glycerol and polyethylene glycol and polyethylene glycol to optimize the liquefaction process using various solvents. The effect of temperature and liquefaction reaction time on wood biomass was also investigated. Based on the value of the hydroxyl number, water content, degree of biomass conversion, and bio-polyol viscosity, a suitable raw material was selected to obtain polyurethane resins. Polyurethane resins containing 90 wt% of bio-polyol were obtained by a one-step method at NCO/OH ratio 1:1 or 1.2:1.

## 2. Experimental

### 2.1. Materials

Oak and alder wood sawdust were received from the local joinery company. Wood sawdust was milled to the size of 60–750 μm using a laboratory mill. During the liquefaction process, purified waste glycerol (purity = 99%, L_OH_ = 1104 mg KOH/g, H_2_O < 0.5%) from Bio-Chem Sp. z o.o. and poly(ethylene glycol) (PEG400) (L_OH_ = 314 mg KOH/g, incl. H_2_O < 0.3%) from Avantor Performance Materials Poland S.A. (Gliwice, Poland). were used. Petrochemical polyol Rokopol^®^M6000 which is polyoxyalkylene triol based on glycerol (L_OH_ = 28 mg KOH/g, H_2_O < 0.1%) was supplied by PCC Rokita (Gliwice, Poland). Petrochemical polyol was used to reduce the viscosity of the polyol mixture during the synthesis of materials. The liquefaction process was catalyzed by a 95% sulfuric acid solution in water obtained from Avantor Performance Materials Poland SA (Gliwice, Poland). As an isocyanate component, a commercially available product named SUPRASEC^®^ 2085 (4,4′-methylene diphenyl diisocyanate-p-MDI) supplied by the Huntsman company was used. Isocyanate contained about 30.5 wt% of free isocyanate groups and average functionality was about 2.9. As a catalyst during polyurethane synthesis dibutyltin dilaurate (DBDT) provided by Sigma-Aldrich (Saint Louis, MO, USA) was used.

### 2.2. Liquefaction of Oak and Alder Wood Sawdust

To prepare the oak and alder wood sawdust for the synthesis both substances were dried at T = 105 °C for 24 h. During liquefaction of oak/alder wood sawdust as a liquefaction solvent glycerol (OG/AG), poly(ethylene glycol) with molar weight 400 g/mol (PEG 400) (OP/AP), and 1:1 (wt. by) mixture of glycerol and poly(ethylene glycol) (OGP/AGP) were used. The mass ratio of solvent (450 g) to biomass (45 g) was 10:1. During the process 3 wt% (13.5 g) of catalyst (sulfuric acid) was added to the reaction mixture. The liquefaction was carried out in a three-neck flask (500 mL). The reactor was equipped with a mechanical anchor stirrer, reflux condenser, and thermocouple. The reaction was carried out at three temperatures—120, 150, and 170 °C for 6 h. Every hour, approximately a 5 mL sample of polyol was collected to determine the water content in a sample. After that polyols were neutralized using a 67% water solution of potassium hydroxide (KOH) and dried at 120 °C under vacuum for 2 h. Sample coding for bio-polyols is summarized in Table 1.

### 2.3. Manufacturing of Polyurethane Resins with High Bio-Polyol Content

During the synthesis one-step method from a two-component system was used for the manufacturing of polyurethane resins. Resins were manufactured at 1:1 and 1.2:1 NCO:OH ratios. As a component, a mixture of obtained biopolyols, Rokopol^®^ M6000, and catalyst (0.5% by weight of DBDL) was used. The amount of bio-polyol in a polyol mixture for AG, OG, AP, and OP was 90% by weight, and for OGP and AGP 80% by weight. Component A was mixed with pure p-MDI (component B) and then homogenized mixture was poured into approximately 2 mm thick mold. After that, the mold was placed in a pre-heated hydraulic and material was hot-pressed at 100 °C for 15 min (5 MPa). To slow down the polymerization reaction of OP/AP samples, 2% by weight of orthophosphoric acid (H_3_PO_4_) was added to the mixture. Sample coding for polyurethane resins is summarized in Table 2.

### 2.4. Determination of Polyols Properties

Determination of the hydroxyl value of the polyols was in accordance with PN-EN 1240:2011 standard. VOLTCRAFT pH 100 ATC pH meter was used to determine the titration point of the dark brown liquid. The hydroxyl value was calculated using Equation (1):

(1) $$\mathrm{HV}=\frac{\left(B-A\right)\cdot M\cdot 56.1}{m}$$

where: A is the volume of 0.5 M KOH required for the titration of a sample (mL), B is the volume of KOH required for the titration of the blank solution (mL), M is the normality of the titrant, and m is the mass of the polyol sample (g).

To determine the biomass conversion, a 1 g sample of polyol was dissolved in ethanol and stirred on a magnetic stirrer for 4 h. Next, the sample was filtered under vacuum and the residues were washed with ethanol. The filter with the remaining solid fraction was dried at 105 °C. The biomass conversion ratio was calculated according to Equation (2):

(2) $${\%\mathrm{Biomass}}_{\mathrm{conversion}\;\mathrm{ratio}}=100-\frac{\left(\frac{w_{1}-w_{2}}{w_{3}}\right)\cdot w_{4}\cdot 100}{w_{5}}$$

where: w_1_ is the total dry weight of filtering crucible with biomass residue; w_2_ is the net weight of filtering crucible without biomass residue; w_3_ is the weight of bio-polyols weighted for biomass conversion analysis; w_4_ is the total weight of bio-polyols obtained via liquefaction, and w_5_ is the weight of biomass added during liquefaction.

The water content of the bio-polyols during and after the process was determined by Karl-Fischer titration. The polyol samples were diluted with methanol and titrated with Fischer reagent. The Karl-Fischer reagent used for the NIST (National Institute of Standards and Technology) volumetric measurements contained a methanol solution of imidazole and sulfur dioxide as the organic base.

The viscosity of bio-polyols at 30 ± 0.5 °C was determined according to ASTM D 4878-08 standard. To define this parameter, an R/S Portable viscometer equipped with a small sample adapter, temperature probe, and temperature control unit was used.

Fourier-transform infrared spectroscopy (FT-IR) was performed to determine the structure of the bio-based polyol and polyurethanes. The analysis was performed using Nicolet 8700 apparatus at a resolution of 4 cm^−1^. This device was equipped with a snap-Gold State II, which allows for making measurements in the reflection configuration mode.

### 2.5. Characterization of Polyurethanes

The thermogravimetric analysis was performed using Netzsch TG 209 apparatus (Netzsch, Selb, Germany). During the test, 5 mg samples were examined in temperature from 50 to 600 °C under a nitrogen atmosphere. The heating rate was 10 °C/min.

Dynamic mechanical analysis (DMA) was conducted to determine the glass transition temperature (T_g_) of polyurethanes. The tests were carried out on DMA Q800 (TA Instruments, Eschborn, Germany). The tests were carried out in flexural mode (single cantilever) with a frequency of 10 Hz and an oscillation amplitude of 10 μm. The temperature of the test was from −100 to 120 °C with a heating rate of 2 °C/min. The dimension of the samples was 40 mm × 10 mm × 4 mm.

## 3. Results and Discussion

### 3.1. Characterization of Bio-Based Polyols Properties

**Figure 1:**
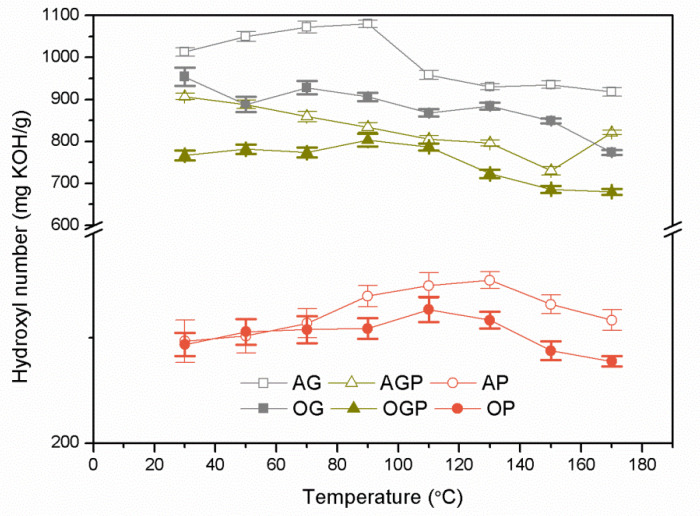
Changes in hydroxyl number depending on the liquefaction reaction temperature during heating.

During the process, dehydrogenation, solvent condensation, or thermal oxidation reactions occur between the solvent and the liquefied wood biomass, which reduces the hydroxyl number. [19,27]. Figure 1 shows the dependence of the hydroxyl number on temperature during heating of a mixture of 10% of biomass (alder and oak wood) with various solvents (90%). There is no clear reaction start temperature for all samples this temperature ranges from 70 to 110 °C. In each case, bio-polyols derived from alder wood, show higher hydroxyl value compared to oak tree biomass. This may result from the composition of wood biomass. Based on the research of Demirbas [28], the alder sawdust contains 44.9% cellulose, 18.6% lignin, 29.8% hemicellulose, and 6.7% moisture content. Derbyshire and Jagtoyen [29] showed that oak sawdust is composed of 53.2% cellulose, 24.6% lignin, and 24.7% hemicellulose. During the condensation reaction, each lignocellulose component is broken down into smaller reactive compounds. For example, cellulose is hydrolyzed to glucosides, levulates, and aldehydes [30]. Two solvents were used in the work, whose content in the bio-polyol was 90%, so the hydroxyl values are determined by the type of solvent used. For AP and OP bio-polyols, the hydroxyl number is significantly lower in comparison to other bio-polyols. This is due to the properties of polyethylene glycol (PEG 400) which hydroxyl number is 200–400 mg KOH/g [31,32].

Table 3 summarizes the final hydroxyl number and biomass conversion of synthesized bio-polyols. At 120 °C for each sample, the liquefaction process resulted in minimal reaction, as evidenced by the high hydroxyl value and low biomass conversion. The opposite relationship was observed for the highest temperature of 170 °C, which was not favorable for processing parameters because the viscosity of bio-polyols increased significantly and the product was not homogeneous. At higher temperatures during the liquefaction process, the solvent condensation reaction predominates [33]. AGP and OGP bio-polyols obtained at 150 °C have a hydroxyl number of 643 and 532 mg KOH/g, respectively. Moreover, a high degree of biomass conversion for both polyols were obtained (97% for AGP and 94% for OGP). This is an advantage for further use in the polyurethane materials industry. Zhang et al. obtained similar properties in their work [34].

**Figure 2:**
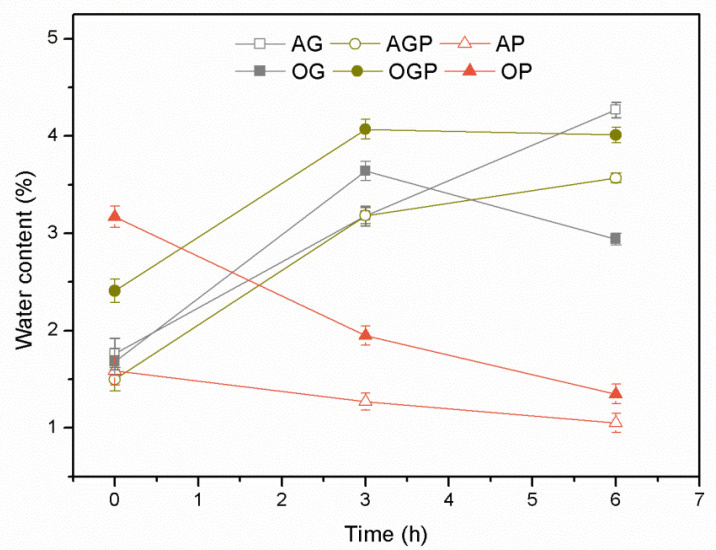
Water content in bio-polyols depending on the time of the liquefaction process.

Figure 2 shows the change in water content of bio-polyols during the liquefaction process at 150 °C depending on the solvent used. It is observed that as the liquefaction process continues, the water content of bio-polyols increases. This effect may indicate the formation of water as a by-product. These results are in line with those obtained by Wang et al. [35]. Initially, the water content in bio-polyols is in the range of 1.5–3.1%, then in the case of bio-polyols with 90% glycerine and a mixture of glycerine and polyethylene glycol increases twice. In the final liquefaction stage after 6 h, the highest water content is 4.3% and 4.0% for AG and OGP, respectively. This indicates a high reaction yield. For bio-polyols containing 90% polyethylene glycol, a decrease in water content was observed in both biomass cases, which indicates the aforementioned solvent condensation reactions.

All polyols, due to the catalyst used-sulfuric acid, had a pH in the range of 1–2. High water content and such strongly acidic pH are detrimental to polyol compounds to be a component of polyurethane materials. For this purpose, all bio-polyols were neutralized with KOH and dried under reduced pressure. The final products after neutralization and drying had a pH in the range of 6.5–7.5 and a water content varies between 0.5–1.2%.

One of the most important properties verifying the possibility of using bio-polyols in industrial processes is their rheological behavior. Rheological measurements may enable the determination of polyols viscosity, shear stress, and fitting polyol behavior to suitable mathematical models.

**Figure 3:**
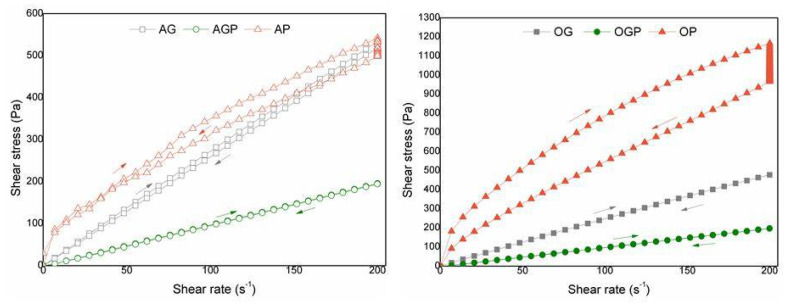
Bio-polyols flow curves.

Figure 3 shows the flow curves for the obtained bio-polyols at the temperature of 150 °C for various solvents used in the liquefaction of alder and oak sawdust. The dependence of the stress function on the shear rate provides information about the nature of the liquid. There are two basic Newtonian and non-Newtonian behaviors. Nonlinear flow fluids are described as non-Newtonian liquids according to Equation (3) [36]:

(3) $$\tan\alpha =\eta_{0}$$

The tangent of the slope of the initial angle of the flow curve relative to the x-axis is closely related to the dynamic viscosity of the liquid. In the case of bio-polyols derived from oak sawdust biomass, an increase in tangent α was observed, which indicates a higher viscosity of the analyzed bio-polyols. The maximum value of shear stress was observed for OP bio-polyol (1166 Pa). However, for the same solvent (PEG400) but different biomass (alder wood), the shear stress for sample AP reduced to 542 Pa·s. A similar relationship was shown in our previous work [31].

The effect when the shear stress of a liquid irreversibly decreases over time at a constant shear rate is called thixotropy. For non-Newtonian liquids, we observe hysteresis, which results from the properties of shear-thinning liquids that return to their original viscosity only with a delay when the shear force stops. This is caused by the progressive breakdown of the fluid structure [37]. Hysteresis loops were obtained during the measurement of the shear stresses for a controlled shear rate from 1 to 200 s^−1^ and from 200 to 1 s^−1^. The smallest hysteresis loops were obtained for AGP and OGP, which proves the smallest thixotropic phenomenon, which is beneficial in industrial processes.

**Figure 4:**
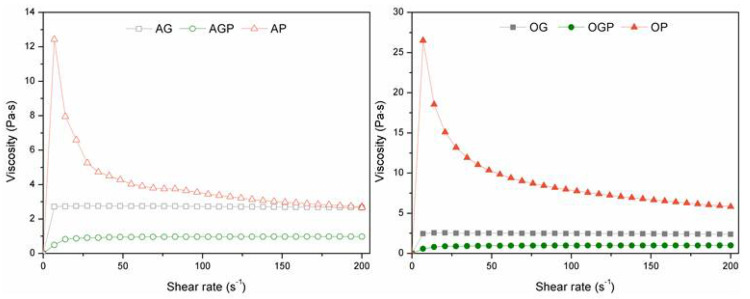
Bio-polyols viscosity curves.

Figure 4 shows the viscosity curves of the obtained bio-polyols. The decrease in viscosity for AP and OP can be attributed to the greater degradation of lignin in the poly(ethylene glycol). Moreover, it can be due to the intensive condensation process, which was confirmed in another work [38]. This may also result from the difference in viscosity of pure solvents (η_PEG400_ = 0.091 Pa·s; η_glycerol_ = 0.978 Pa·s). Furthermore, during the reaction more and more macromolecules dissolve in the solvent, which may result in increased viscosity. This can be noticed for AGP and OGP bio-polyols.

To assess whether the bio-polyols obtained are pseudoplastic liquids, an analysis of mathematical models (Ostwald–Waele, Herschel–Bulkley, and Bingham) can be used [39]. In this work, the Herschel–Bulkley model was used. This model is described by the following formula (4), where τ is the shear stress, τ_0_ is the yield stress, γ is the shear rate, K is flow consistency, and n is flow behavior index.

(4) $$\tau =\tau_{0}+{K\gamma }^{n}$$

The Herschel–Bulkley linear functions based on the rheological data from bio-based polyol is presented in Table 4. Based on the obtained data, it can be concluded that bio-polyols obtained during liquefaction of alder and oak wood biomass were Newtonian and non-Newtonian fluids. AP and OP samples were recognized as non-Newtonian fluids. The flow exponent for OP and AP bio-polyols is significantly lower than 1 and therefore these polyols can be classified as shear thinned liquids.

### 3.2. FTIR Analysis of Bio-Polyols

**Figure 5:**
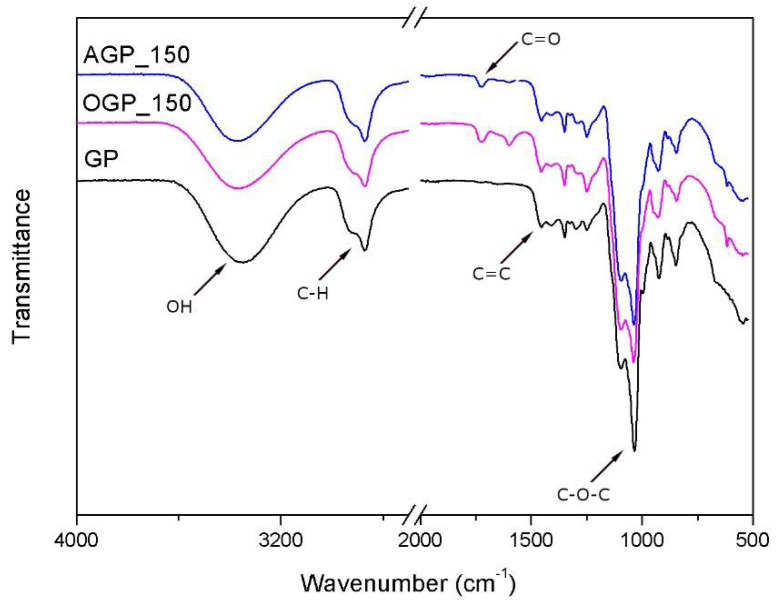
FTIR spectra of bio-polyols.

FTIR analysis was performed to examine the characteristics of liquefied polyol from alder and oak wood. Figure 5 shows the spectrum of solvents and two bio-polyols AGP and OGP obtained at a process temperature of 150 °C. Intense peaks at 3365–3350 cm^−1^ are from vibrations of hydroxyl groups. Most of the signals received in the spectrum coincide with signals coming from the solvents due to their 90% content in the polyol. In the 2870–2930 cm^−1^ range, the C-H stretching modes of the aliphatic CH_3_, CH_2_, and CH groups are observed [40]. New bands from OGP bio-polyol were observed. The peak at 1722 cm^−1^ may correspond to C=O bonds and the peak at 1598 cm^−1^ may be related to C=C bonds. Both signals may come from fatty acids and hemicellulose present in the structure of bio-polyols [41]. It can also be concluded that the band at 1722 cm^−1^ may be related to the presence of levulinians in the polyol sample [42,43]. Absorbance bands at 1455 and 1350 cm^−1^ result from aromatic skeletal vibrations combined with the symmetrical bending mode of hydroxymethyl present in cellulose [44]. Phenolic hydroxyl groups from the phenylpropane structure present in lignin that reacted in the liquefaction process with the solvent are visible at 1250 cm^−1^ [45,46]. The intense peak at 1090–1035 cm^−1^ was characteristic of vibrations of the C-O-C ether group derived from the solvent [47]. Signals at 845–920 cm^−1^ were attributed to C=C and C-H bond vibration in the aromatic rings present in the biomass [48].

### 3.3. Thermal Properties of Polyurethane Resin

**Figure 6:**
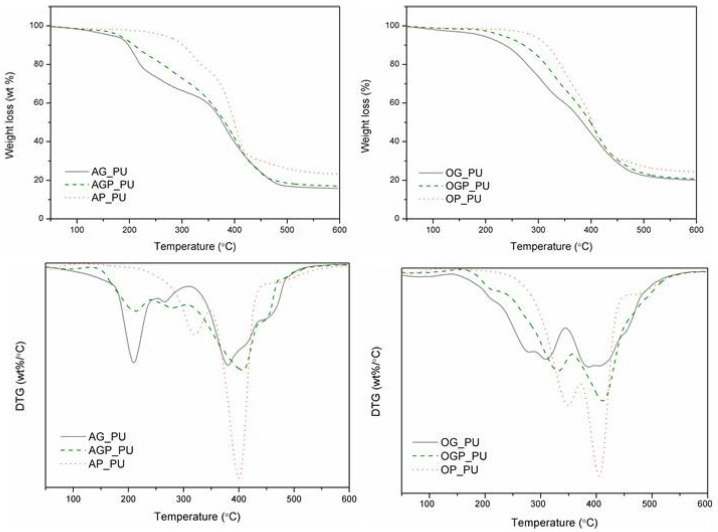
The TGA and DTG curves of the polyurethane resins.

Figure 6 presents the thermogravimetric curves of the polyurethane resins with an NCO:OH ratio of 1:1 and its value is tabulated in Table 5. Polyurethanes are widely known as relatively thermally unstable materials. This is due to the presence of urethane bonds in their structure. Dissociation of urethane bonds is based on three main mechanisms: dissociation to isocyanates and alcohol, formation of primary amines, olefins, and formation of a secondary amine [49]. Thermal dissociation of urethane bonds (200 °C), and urea bonds (250 °C) is followed by breaking of isocyanurate bonds and free NCO at (350 °C) [18]. The first degradation step of the investigated resins occurred between 240 °C and 310 °C which can be attributed to the degradation of urethane bonds. The second degradation stage was observed in the range between 320 °C and 470 °C. This stage results from the disruption of polymer chains in bio-polyols such as poly(ethylene glycol) [50], cellulose or lignin derivatives [50,51,52], breaking of isocyanurate bonds, and free NCO. For resins containing glycerol in their composition (AG_PU, AGP_PU, OG_PU, and OGP_PU), the formation of urea groups with pMDI does not proceed at an even rate. Similar changes were observed during the degradation of PU derived from vegetable oil. The similarity to the obtained bio-polyols results from the structure of vegetable oil-based polyols, where we have a glycerol skeleton and fatty acid side chains [8,53]. The highest thermal stability was demonstrated by AP_PU and OG_PU, which may be due to the greater content of high molecular weight PEG 400 [7].

**Figure 7:**
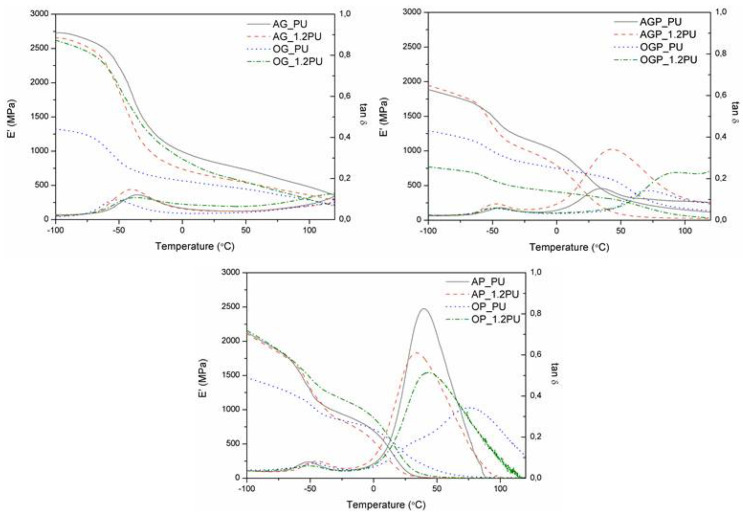
*Tan δ* and storage modulus (E′) of polyurethane resins.

Figure 7 shows the storage modulus (E′) and loss tangent (tan δ) curves for the obtained polyurethane resins. The phase transition from the glassy state to the rubbery state is visible for resins containing only glycerol as solvent. In the case of other materials, a double transition is observed, indicating distinct areas of lower and higher crosslinking density. A single tan δ peak indicates a homogeneous internal molecular structure in which stress concentration can be avoided and material can absorb more energy as a result of loading. A double tan δ peak in polyurethane resins may be due to phase separation which leads to the heterogeneous molecular structure [16]. This effect may be explained by the higher complexity of the bio-polyols used to obtain the materials, which leads to non-uniform cross-link density. The peak at lower temperatures is caused by the thermal mobility of the soft segments. It starts at −85 °C with a peak at −35 °C for AG_PU and −42 °C for OG_1.2PU, while for OG_PU it is −56 °C and −36 °C for OG_1.2PU. The second main peak for the remaining resins results from the cross-linking points derived from lignocellulosic biomass and the thermal mobility of rigid segments. These segments consist of urethane bonds [10]. The phase transitions shown are clear, which proves the high thermal activity [16]. Data are presented in Table 6.

Based on the data in Table 6, it was observed that all PU resins with an NCO:OH ratio of 1:1 have a higher modulus (E′). It is because a higher NCO:OH ratio causes the formation of micropores, which can reduce the stiffness of the material.

## 4. Conclusions

During this study, the optimization of temperature and liquefaction time was performed to obtain liquefied bio-polyols based on hardwood species with minimal residues. The physical and chemical properties of bio-polyols were analyzed and compared. The reaction time of 6 h and a temperature of 150 °C were estimated as the optimal parameters for the production of bio-polyol ensuring a high liquefaction yield (65–97%) with a suitable hydroxyl number (215–770 mg KOH/g). The results reported here confirm that liquefaction of alder and oak wood waste can be successfully applied in the production of bio-polyols. Furthermore, these polyols can be used for polyurethane materials manufacturing. The polyurethane resins were obtained using up to 90 wt% bio-polyol in a polyol mixture. The obtained materials were characterized by and high elastic modulus and due to their beneficial properties, they can be used indoor and outdoor as a material for decking. Understanding the liquefaction process, yield of the reaction, and structure of bio-polyols can be beneficial in the production of PU materials.

## Figures and Tables

**Table 1:** An explanation of the abbreviations used to describe polyols samples.

Sample Name	Biomass	Solvent
AG	Adler wood	glycerol
AGP		glycerol: poly(ethylene glycol)
AP		poly(ethylene glycol)
OG	Oakwood	glycerol
OGP		glycerol: poly(ethylene glycol)
OP		poly(ethylene glycol)

**Table 2:** An explanation of the abbreviations used to describe polyurethane materials.

Sample Name		NCO:OH
AG_PU	OG_PU	1:1
AGP_PU	OGP_PU	
AP_PU	OP_PU	
AG_1.2PU	OG_1.2PU	1.2:1
AGP_1.2PU	OGP_1.2PU	
AP_1.2PU	OP_1.2PU	

**Table 3:** The hydroxyl value and biomass conversion of liquefied bio-polyols.

Sample	Reaction Temperature (°C)	Hydroxyl Number (mg KOH/g)	Biomass Conversion (%)
	120	815	68
AG	150	770	92
	170	612	82
	120	723	82
AGP	150	643	97
	170	320	98
	120	324	70
AP	150	238	80
	170	127	87
	120	813	68
OG	150	736	95
	170	709	92
	120	732	90
OGP	150	532	94
	170	352	97
	120	267	59
OP	150	215	65
	170	114	72

**Table 4:** The Herschel–Bulkley linear functions based on the rheological data from bio-based polyol.

Bio-Polyol Symbol	The Herschel–Bulkley Linear Functions				
	Function	τ_0_ [Pa]	K [Pa·s^n^]	n [−]	R^2^
**AG_150**	y = 2.7857·x^0.9891^	0	2.7857	0.9891	0.9982
**AGP_150**	y = 0.8171·x^1.0339^	0	0.8171	1.0339	0.9996
**AP_150**	y = 33.6707 + 9.8791·x^0.7356^	33.6707	9.8791	0.7356	0.9839
**OG_150**	y = 2.6809·x^0.9753^	0	2.6809	0.9753	0.9968
**OGP_150**	y = 0.8246·x^1.0343^	0	0.8246	1.0343	0.9998
**OP_150**	y = 81.1781 + 15.8788·x^0.7344^	81.1781	15.8788	0.7344	0.8638

**Table 5:** Characteristics of thermal degradation of the polyurethane resins.

Sample Name	Mass Loss (wt%)			Residue at 600 °C (wt%)	T_max1_	T_max2_
	2	5	10			
	Temperature (°C)					
AG_PU	108	165	197	15.8	209	380
AGP_PU	121	180	208	17.0	214	406
AP_PU	185	265	301	23.2	318	399
OG_PU	103	192	236	20.1	308	397
OGP_PU	185	230	272	20.7	329	412
OP_PU	234	289	315	24.4	349	405

**Table 6:** Dynamic mechanical and thermal properties of polyurethane resins.

Sample	T_g_ (°C)	E′ (MPa) at 25 °C
AG_PU	–34.9	839.9
AG_1.2PU	–42.3	651.6
OG_PU	−52.3	505.1
0G_1.2PU	−36.1	671.1
AGP_PU	−43.5	609.8
AGP_1.2PU	−47.1	339.9
OGP_PU	−48.5	665.3
OGP_1.2PU	−49.7	351.8
AP_PU	−51.8	114.9
AP_1.2PU	−47.9	62.4
OP_PU	−45.2	358.4
OP_1.2PU	−54.6	232.6

## Data Availability

Data sharing is not applicable to this article.
